# Fast phage detection and quantification: An optical density-based approach

**DOI:** 10.1371/journal.pone.0216292

**Published:** 2019-05-09

**Authors:** Denis Rajnovic, Xavier Muñoz-Berbel, Jordi Mas

**Affiliations:** 1 Departament de Genètica i de Microbiologia, Universitat Autònoma de Barcelona, Edifici C. Campus de Bellaterra, Cerdanyola del Vallès, Barcelona, Spain; 2 Instituto de Microelectrónica de Barcelona (IMB-CNM, CSIC), Cerdanyola del Vallès, Barcelona, Spain; University of York, UNITED KINGDOM

## Abstract

Since 1959 with the proposal of Double Agar Layer (DAL) method for phage detection and quantification, many sophisticated methods have emerged meanwhile. However, many of them are either too complex/expensive or insensitive to replace routine utilization of DAL method in clinical, environmental and industrial environments. For that purpose, we have explored an alternative method for the detection and quantification of bacteriophages that fulfills the criteria of being rapid, simple and inexpensive. In this paper we have developed a method based on the analysis of optical density kinetics in bacterial cultures exposed to phage-containing samples. Although the decrease in optical density caused by cell lysis was one of the first observable consequences of the effect of viral infection in bacterial cultures, the potential of the method for the assessment of phage abundance has never been fully exploited. In this work we carry out a detailed study of optical density kinetics in phage-infected bacterial cultures, as a function of both, phage abundance and initial concentration of the host organisms. In total, 90 different combinations of bacteria/phage concentrations have been used. The data obtained provide valuable information about sensitivity ranges, duration of the assay, percentages of inhibition and type of lysing behavior for each phage concentration. The method described can detect, as few as 10 phage particles per assay volume after a phage incubation period of 3.5h. The duration of the assay can be shortened to 45min at the expense of losing sensitivity and increasing the limit of detection to 10^8^ pfu/ml. Despite using non-sophisticated technology, the method described has shown sensitivity and response time comparable to other high-end methods. The simplicity of the technology and of the analytical steps involved, make the system susceptible of miniaturization and automation for high-throughput applications which can be implemented in routine analysis in many environments.

## Introduction

Methods for the detection and quantification of bacteriophages have been available ever since their discovery by Felix d’Herelle in 1917 [1]. These methods, based on the presence of lysis plaques in lawns of host bacteria growing in a double agar layer (DAL), were described in detail by Mark Adams in 1959 [2] and, with the addition of several modifications and improvements [3–7] they have constituted the workhorse of virus quantification until now.

Despite the well-established value of the DAL method, the long times required to achieve detection (24 to 48 h), the labor intensive nature of the methodology, and the impossibility to convert it to an automated or semi-automated format for high throughput testing, make the classical DAL method ill-suited to provide a response to the challenges of current clinical, environmental or industrial applications. In the clinical field, for example, the need to assess phage interference in microbiological diagnostic tools, both pathogen detection and antibiotic susceptibility testing [8] and the growing need to monitor emerging phage therapy technologies [9–13] call for the development of reliable and fast methods for phage detection. In public health, detection of enteric phages has been proposed as an indicator of fecal contamination in water [14,15]. Finally, the availability of fast phage detection methods in the industrial environment, has been sorely missing for many years. Monitoring of phages responsible for the failure of microbe-based industrial processes such as yogurt or cheese production [16–20], as well as the use of phages in the biocontrol of food pathogenic bacteria or as an aid in the eradication of biofilms [13], all require fast, inexpensive and sensitive methods for routine monitoring applications.

The growing interest in phage monitoring in these fields has prompted the development of a new generation of agile and sensitive methods able to overcome the limitations derived from DAL. These methods are based either on the direct detection of viral particles by PCR [21], qPCR [22, 23], Raman spectroscopy [24], immunoassay [25, 26], MALDI-TOF [27, 28], or on the lysis of the host organism by flow cytometry [29], fluorescence microscopy [30], enzyme release [13, 31, 32], surface plasmon resonance (SPR) [33, 34] or impedance measurements [35]. Sophisticated as they are, many of these methods do not match the sensitivity and precision of the DAL method. Moreover, whereas most of them are considerably faster, the complexity and cost of the instrumentation required for the analysis constitute a definitive barrier for their routine implementation in many environments.

With this in mind, we revisit the idea of using optical density measurements as a simple and inexpensive method for the detection and quantification of bacteriophages in all kind of samples, at different levels of sensitivity and in remarkably short times. Although the decrease in optical density caused by cell lysis was one of the first observable consequences of the effect of viral infection of bacterial cultures, the potential of the method for the assessment of phage abundance has never been fully exploited. In this work we carry out a detailed study calibrating optical density kinetics as a function of both, phage abundance and concentration of the host organisms. Our study determines the percentage of growth inhibition from integrated growth curves and correlates this value to the amount of phage initially present in the sample. The results are discussed in the context of their use in the design of simple and sensitive methods for the monitoring of bacteriophages in industrial, clinical or environmental samples.

## Materials and methods

### Microorganisms and growth conditions

*Escherichia coli* DSMZ 613 (DSMZ, Germany) was grown overnight in Luria-Bertani (LB) medium at 37 °C in an incubator shaker (100 rpm). The cultures were centrifuged at 4000 x *g* for 10 min and resuspended in 1 mL of 0.1 M phosphate buffer (PB, pH = 7.2). Optical density of the cell suspensions was measured at 600 nm using a Smartspec Plus spectrophotometer (Bio-rad, California, USA) and diluted to the required concentration using 0.1 M PB. Bacterial concentrations were determined by viable plate counts and expressed as colony forming unit per mL (cfu/mL).

Bacteriophage T4 was kindly provided by Dr. M. Llagostera from the Department of Genetics and Microbiology of the Autonomous University of Barcelona. Phage lysates were prepared following the protocol of Bonilla et al. [36] using *E*. *coli* as a host. 100 mL of an *E*. *coli* culture growing in LB broth supplemented with CaCl_2_ (1 mM) and MgCl_2_ (1 mM) were infected with 100 μL of virus suspension. After achieving lysis, the culture was centrifuged at 4000 x *g* for 20 min. The supernatant was filtered through a 0.22 μm membrane cellulose acetate filter (Whatman) and further treated with chloroform to remove lipids. The resulting suspension was concentrated by ultrafiltration using Amicon Ultra-15 centrifuge tubes with a cutout size of 100 kDa. Additional endotoxin removal, prior to sample storage, was done using 1-octanol as described by Szermer-Olearnik and Boratyński [37] followed by membrane dialysis in a Spectra/Por Float-A-Lyzer G2 Dialysis Device with a MWCO of 3.5–5 kDa. The purified product was stored in SM buffer [36] at 4 °C. Determination of virus concentration was performed by counting plaque forming unit (pfu) using the double layer agar method described by Adams [2]. Prior to their use, virus suspensions were diluted in LB to achieve the desired final concentration.

### Experimental design

Our objective was to characterize the optical density kinetics of different combinations of phage/bacteria concentrations in order to assess to what extent kinetic measurements could be used as a reliable indicator of the abundance of phage in a certain sample. Therefore, an experiment was designed in which bacterial concentrations ranging from 10^5^ to 5x10^8^ cfu/mL were tested in combination with concentrations of T4 phage ranging from 0 to 5x10^8^ pfu/mL.

Overnight cultures of *E*. *coli* were centrifuged and the pellets resuspended in 0.1 mM PB to achieve a concentration of 10^10^ cfu/mL. The resulting suspensions were subject to serial dilution in such a way that after mixing with the phage in LB medium the desired final concentration was obtained. In a similar way, stock lysates of T4 were serially diluted in LB medium in order to achieve the desired concentrations. For each assay, 160 μL of LB were mixed with 20 μL phage solution, 20 μL of bacteria solution and 20μL of PB in transparent 96-well plates (Thermo Scientific, Massachusetts, USA). The plates were incubated at 37 °C in a Varioskan Flash plate reader (Thermo Scientific, Massachusetts, USA) and OD_600_ was recorded at regular intervals. Samples, controls and blanks were always assayed as triplicates.

### Analysis of the experimental data

**Fig 1:**
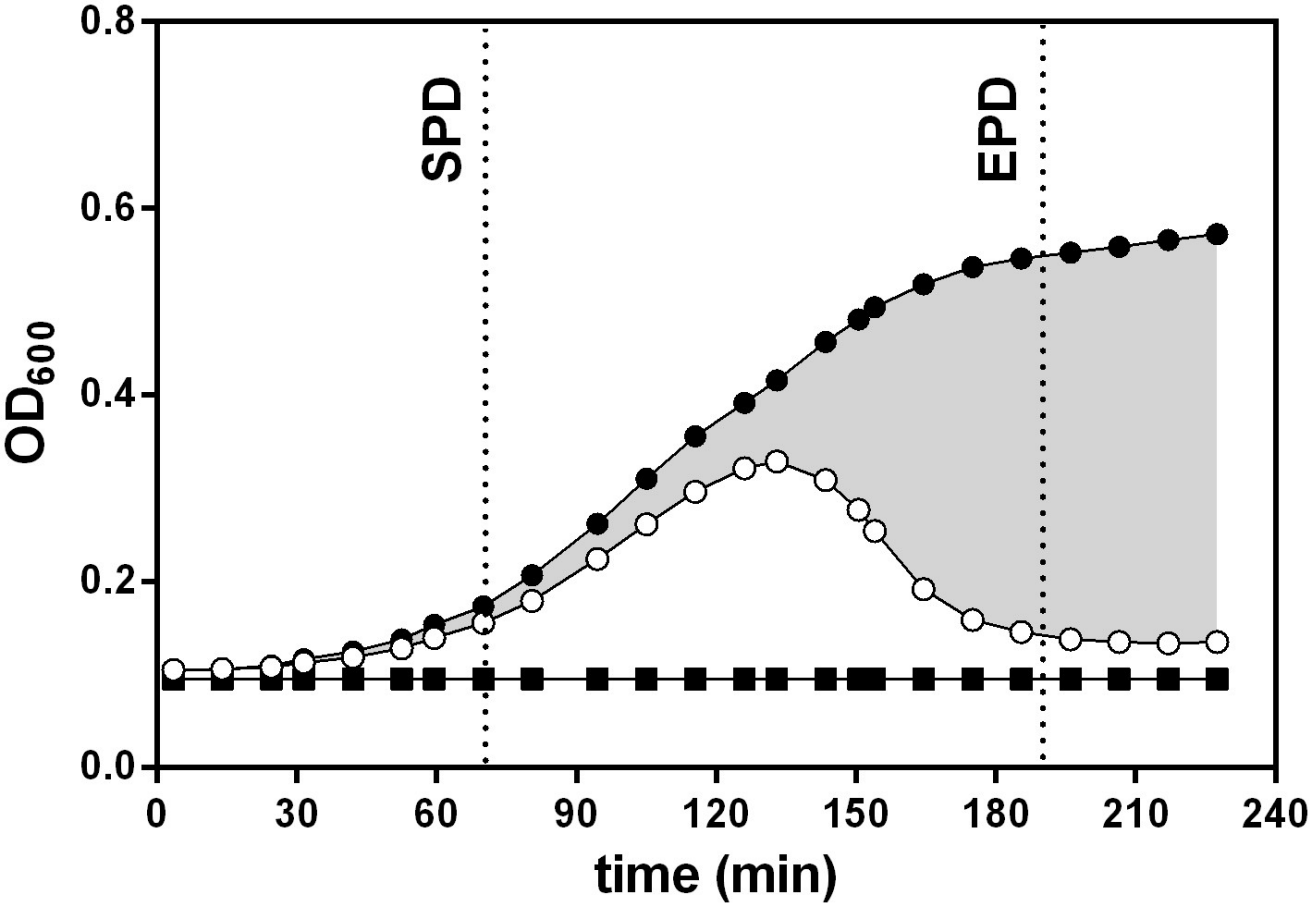
Graphical representation of the procedure used to determine inhibition due to phage lysis in each experiment. Optical density vs time curves of a control (●) and a phage-inoculated culture (○) were integrated and subtracted. The difference, represented by the shaded area, indicates the extent of the inhibition. This area is expressed as a percentage of the area of the control. In cases with little or no phage effect, the shaded area is very small and the percentage of inhibition approaches 0%. In the most extreme cases the shaded area virtually coincides with the area of the control, and the percentage of inhibition approaches 100%. In order to standardize all calculations, integration is carried out between the Start Point of Detection (SPD) and End Point of Detection (EPD) as defined in the text.

The experimental design used provides an extensive set of data that has to be further processed in order to carry out a proper interpretation of the results. For each bacteria concentration used we calculated the Start Point of Detection (SPD) as the time required for the different controls (bacteria without phages) to reach the threshold of detectable growth. We arbitrarily defined this threshold as a growth rate of 0.002 OD units per min. For further calculations we also defined the End Point of Detection (EPD) as a time corresponding to SPD + 120 min, thus allocating a 2-hour window for the assay to develop (Fig 1).

### Growth inhibition due to lysis

For each bacteria/phage combination, we integrated the area of the curve between the points SPD and EPD. Numerical integration was carried out using the Euler method with the sampling interval as the integration step. The integrated areas were used to calculate a percentage of inhibition (PI) using the following formula based on the procedure described by Xie et al [11]:

$$PI\;=\;\frac{{(A}_{control}-A_{blank})-(A_{phage}-A_{blank})}{\left(A_{control}-A_{blank}\right)}∙100$$ (1)

in which *A*_*control*_ corresponds to the area of the curve of a control culture without phage inoculation, *A*_*phage*_ corresponds to the area of the curve of a culture exposed to a certain phage concentration, and *A*_*blank*_ corresponds to the area of the baseline curve consisting only of culture medium without either bacteria or phages (Fig 1). Simplification of Eq 1 yields:

$$PI\;=\;\frac{A_{control}-A_{phage}}{A_{control}-A_{blank}}∙100$$ (2)

As a rule, in the absence of phage lysis, PI equals 0% and complete lysis gives a PI of 100%. Intermediate results can be correlated to phage concentration for each bacterial concentration used.

### Probability of void samples

The probability of void samples (samples containing no phages) was calculated using the probability mass function of the Poisson distribution [38] expressed as follows:

$$P(x\;=\;N)\;=\;\frac{{\left(c∙V\right)}^{N}∙e^{-c∙V}}{N!}$$ (3)

Where *N* is the number of phages expected (in this case 0), *c* is the concentration of phages in the medium subject to sampling and *V* is the volume of the sample. For the specific case of *N* = 0, Eq 3 can be simplified to:

$$P(x\;=\;0)\;=\;e^{-c∙V}$$ (4)

## Results and discussion

**Fig 2:**
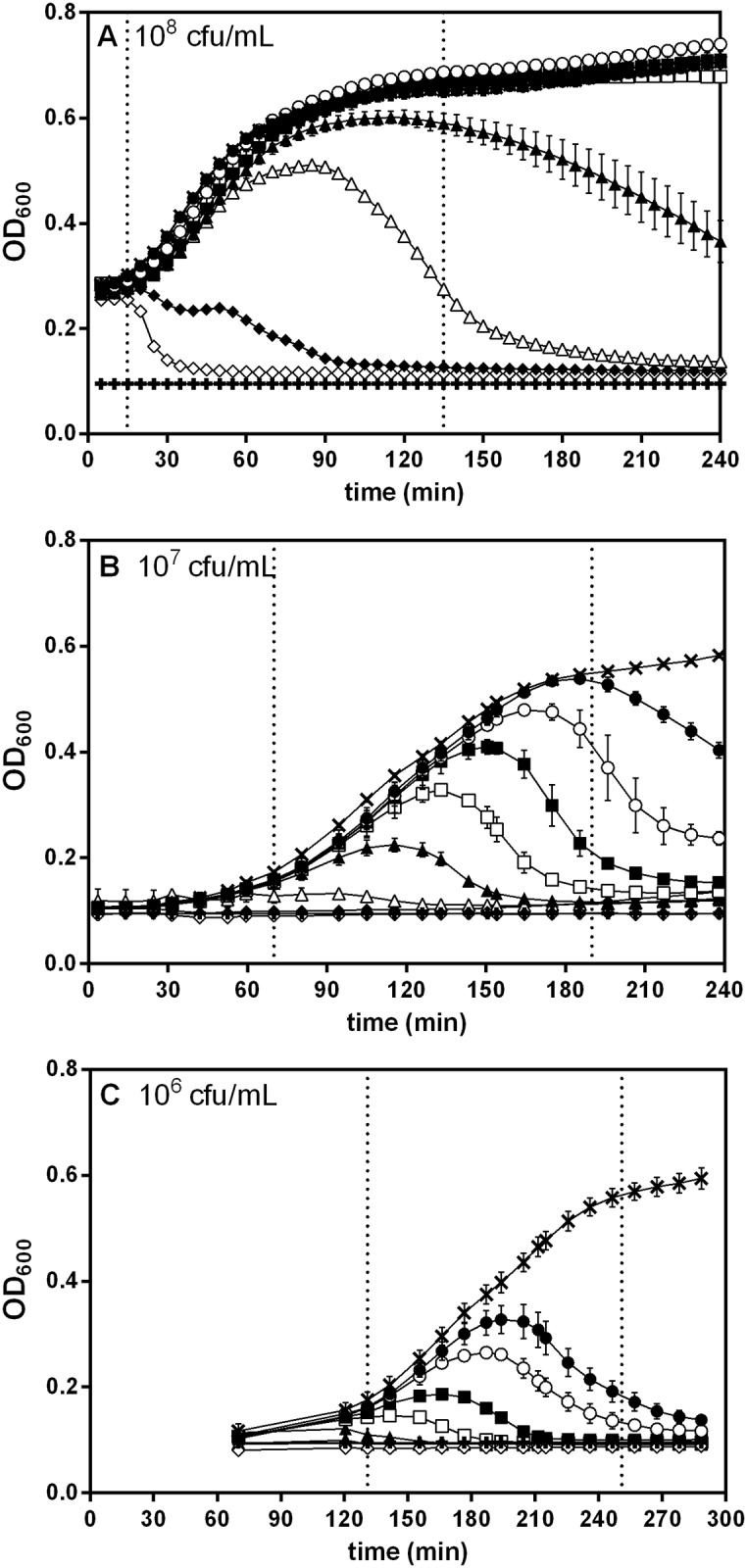
Evolution of optical density during time in cultures of *E*.*coli* exposed to different T4 phage concentrations. Several bacteria concentrations: **A**) 10^8^, **B**) 10^7^ and **C**) 10^6^ were tested against different phage concentrations: 5x10^8^ (◇), 5x10^7^ (◆), 5x10^6^ (△), 5x10^5^ (▲), 5x10^4^ (□), 5x10^3^ (■), 5x10^2^ (○), 5x10^1^ pfu/ml (●) and without phages (×). Error bars represent the standard deviation (n = 3).

In order to check the suitability of optical density measurements for the detection of low phage concentrations we carried out a series of experiments in which different concentrations of bacteria (10^5^, 5x10^5^, 10^6^, 5x10^6^, 10^7^, 2.5x10^7^, 5x10^7^, 10^8^, 2.5x10^8^ and 5x10^8^ cfu/mL) were exposed to different concentrations of phage (0, 5x10^1^, 5x10^2^, 5x10^3^, 5x10^4^, 5x10^5^, 5x10^6^, 5x10^7^, and 5x10^8^ pfu/mL). Each combination of phage/bacteria was incubated at 37 °C and optical density at 600 nm was recorded at regular intervals. In total, 90 different combinations of bacteria/phage concentrations were used. Representative results corresponding to three bacterial concentrations (10^8^, 10^7^ and 10^6^ cfu/mL) have been represented in Fig 2. The remaining data can be found in S1 Fig and S1 Dataset. Fig 2A shows the evolution of optical density over time for a 10^8^ cfu/mL *E*. *coli* culture exposed to different initial phage concentrations. As can be seen, optical density of the control increased during the first 90 minutes until the culture reached stationary phase. The effect of phage addition depended to a large extent on the concentration of phage. Addition of 5x10^8^ pfu/mL resulted in a very fast decrease in optical density: after only 25–30 minutes, the culture was completely lysed and optical density had reached the level of the blank. Lower phage concentrations, however, had a less pronounced effect. Thus, 10^7^ pfu/mL gave rise to a small decrease in optical density during the first 30 minutes, followed by a second decrease 30 minutes later that brought the culture down to blank levels. This stepwise behavior is highly consistent with the expected kinetics of the lytic cycle for phage T4 which has a latent period of 21 to 35 minutes [39]. With lower phage concentrations (10^6^ and 10^5^ pfu/mL) cultures grew to some extent before lysis was apparent. Specifically, at 10^6^ pfu/mL optical density started to decrease 90 minutes after the beginning of the experiment while at 10^5^ pfu/mL this decrease was observed only after 120 minutes of incubation. Below 10^5^ pfu/mL (5x10^4^, 5x10^3^, 5x10^2^ and 5x10^1^ pfu/mL), phage addition had virtually no effect on the kinetics of the culture and the increase in optical density was not much different from that observed in the control.

When the same experiment was performed using a tenfold lower cell concentration (10^7^ cfu/mL) (Fig 2B) the results were somewhat different. While in the 10^8^ cfu/mL culture of Fig 2A optical density started to increase after only 15 minutes of incubation, in this case, OD increase started 70 minutes after the beginning of the experiment. The control without phages showed unrestricted growth which slowed down after 180 minutes. As before, addition of phages had a clear impact on growth dynamics. Even very low amounts of phage (5x10^1^ pfu/mL) caused detectable cell lysis, with a decrease in OD starting at 190 minutes. Addition of higher phage concentrations shortened the time required for the onset of detectable lysis. That is, the time necessary to detect cell lysis at 5x10^2^, 5x10^3^, 5x10^4^ and 5x10^5^ pfu/mL was progressively shortened from 190 to 133 minutes. A regular trend seems apparent when looking at this data, in which the time required to reach the onset of lysis increased by roughly 20–25 minutes every time that phage concentration was decreased one order of magnitude. Cultures containing phage concentrations above 5x10^5^ pfu/mL did not grow and their OD remained constant over time, indicating that bacterial populations lysed before having the chance to reach detectable OD levels.

Finally, Fig 2C shows the kinetics of OD for a 10^6^ cfu/ml *E*. *coli* culture exposed to the same phage concentrations as above. In this case OD in the cultures only started to increase after 131 minutes. As in the other cases, the control without phages grew unrestricted, but the addition of as little as 5x10^1^ pfu/mL at the beginning of the experiment resulted in the lysis of the culture, with OD starting to decrease at 199 minutes. As before, higher phage concentrations (5x10^2^ and 5x10^3^ cfu/mL) resulted in lower times to lysis (185 and 166 minutes). Increasing phage concentration above these values resulted in very low or null increases in optical density, once more indicating that the culture had been lysed before having the opportunity to reach a detectable OD level.

Overall, comparison of the three graphs shows several facts: First, decreasing initial cell concentration results in progressively longer lag periods before growth and/or lysis can be detected using optical density. In Fig 2A (10^8^ cfu /mL) changes can already be observed 20 minutes after the start of the experiment. When 10^7^ cfu/mL are used (Fig 2B) this lag extends to 60 minutes. Use of 10^6^ cfu/mL (Fig 2C) further extends this lag to 120 minutes.

**Fig 3:**
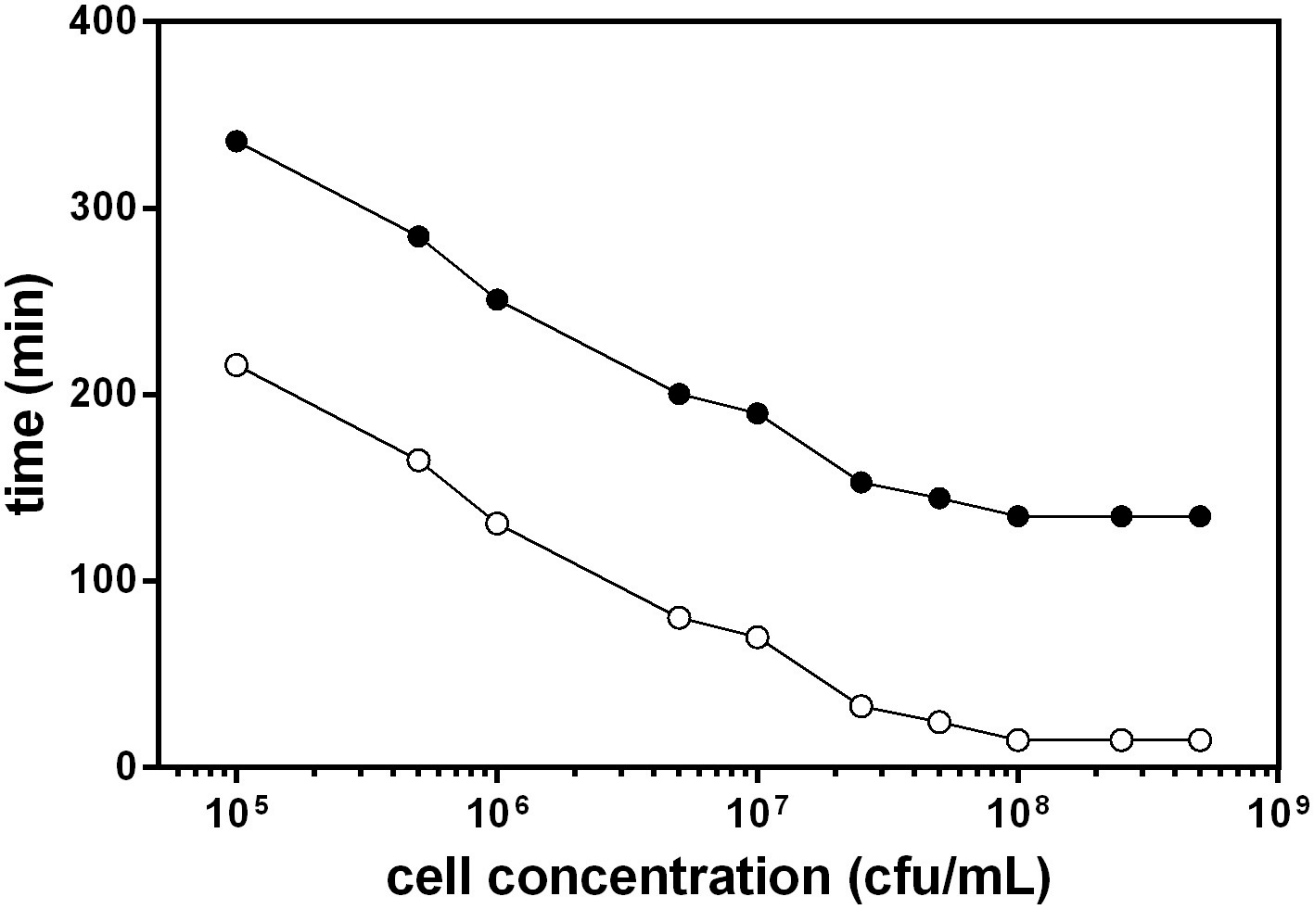
Representation of the Start Point of Detection (SPD) (○) and End Point of Detection (EPD) (●) as a function of the concentration of bacteria used in the experiment. SPD is the time at which detectable growth (defined as ≥ 0.002 OD units per minute) starts. EPD is defined as SPD + 120 minutes, the time usually required to carry out a reliable phage detection.

In order to assess systematically the magnitude of this delay we recorded the time required for the different controls to reach the threshold of detectable growth. We arbitrarily defined this threshold as a growth rate of 0.002 OD units per min. This time, referred to as the Start Point of Detection (SPD) has been plotted in Fig 3 for all the different conditions used, as a function of initial bacterial concentration. As can be seen in Fig 3, the Start Point of Detection decreases exponentially when increasing cell concentration. Thus, at the lowest cell concentration used (10^5^ cells/mL), SPD is 200 minutes. This time decreases at a rate of 70 minutes per log increase in cell concentration, down to approximately 20 minutes. In the same graph, the End Point of Detection (EPD) has also been represented. As explained in the methods section, EPD is calculated as SPD + 120 min and corresponds roughly to the time required to carry out reliable phage detection at each bacterial concentration. EPD values range from a maximum of 5.5 h when using 10^5^ cfu/mL, to 2h 15’ when using higher cell concentrations.

A second observation concerning the experiment presented in Fig 2 refers to the range of phage concentrations that can be detected using each cell concentration. In general, the kinetics of optical density show three types of behavior:

1. **No lysis. No effect on growth**. High bacterial concentrations combined with low phage concentrations result in unrestricted growth that most of the times cannot be differentiated from the growth kinetics of the control. This can be seen in Fig 2A when 10^8^ cfu/mL are exposed to 10^4^, 10^3^, 10^2^ and 10^1^ pfu/mL).
2. **Complete lysis. No growth**. Low bacterial concentrations combined with high phage concentrations display no detectable growth as the complete culture is lysed before optical density starts to increase. This behavior can be observed in Fig 2C, in which 10^6^ cfu/mL exposed to 10^6^, 10^7^ and 10^8^ pfu/mL show virtually no growth.
3. **Delayed lysis**. A detectable increase in OD occurs, but after a certain time, which depends on the concentration of phage, OD starts to decrease as a consequence of bacterial lysis. This can be observed in Fig 2B (10^7^ cfu/mL) when the culture is exposed to 10^1^, 10^2^, 10^3^, 10^4^ and 10^5^ pfu/mL.

**Fig 4:**
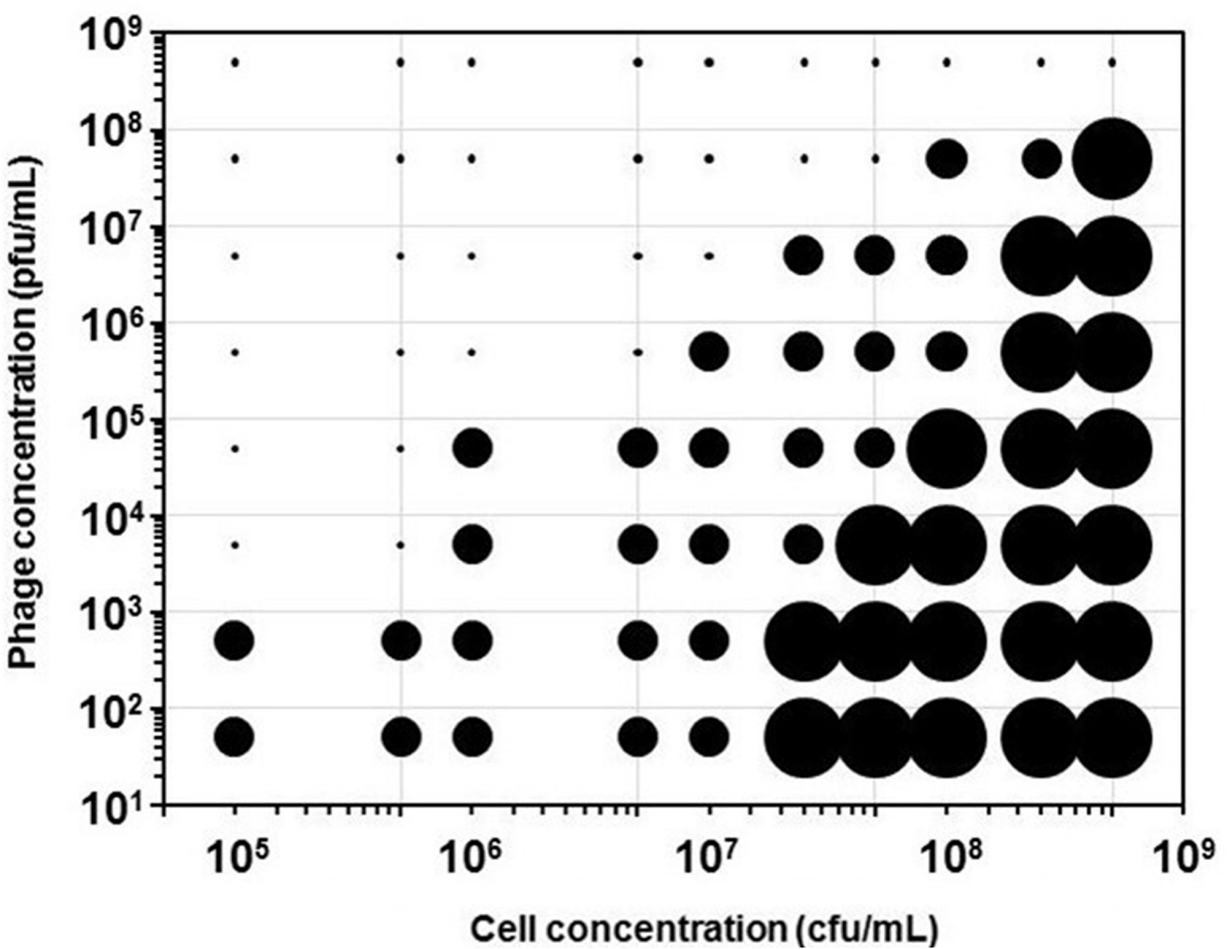
Lysis behavior of the different combinations of T4 and *E*. *coli* concentrations assayed. Large circles indicate the absence of lysis; small dots indicate complete lysis right from the beginning of the experiment. Intermediate circles indicate the existence of delayed lysis, this is, significant bacterial growth can be observed before the onset of lysis.

The type of behavior observed has been recorded for each of the 90 different combinations of phage/bacteria assayed. The results are shown in Fig 4. Data have been encoded in such a way that **Complete Lysis** is represented as a very small dot, **No Lysis** appears as a large size circle, and **Delayed Lysis** is shown as an intermediate sized circle. As can be seen in the right hand side of the graph, cultures with high cell concentrations are not sensitive to low phage numbers as the culture reaches stationary phase before the phage has had time to propagate enough to cause detectable lysis. In opposition, on left hand side of the graph it can be observed how low concentrations of bacteria are completely lysed by phage concentrations of 5x10^4^ pfu/mL or higher. As a rule, decreasing initial cell concentration improves detection at low phage titers, but there is a tradeoff. Use of low cell concentrations, as seen in Fig 3, increases considerably the time required for the assay. In general terms, the best results for phage detection were obtained with the use of 5x10^6^ and 10^7^ cfu/mL. In this concentration range, **delayed lysis** was detected for samples containing only 50 pfu/mL with a short incubation between 2 and 3 hours.

**Fig 5:**
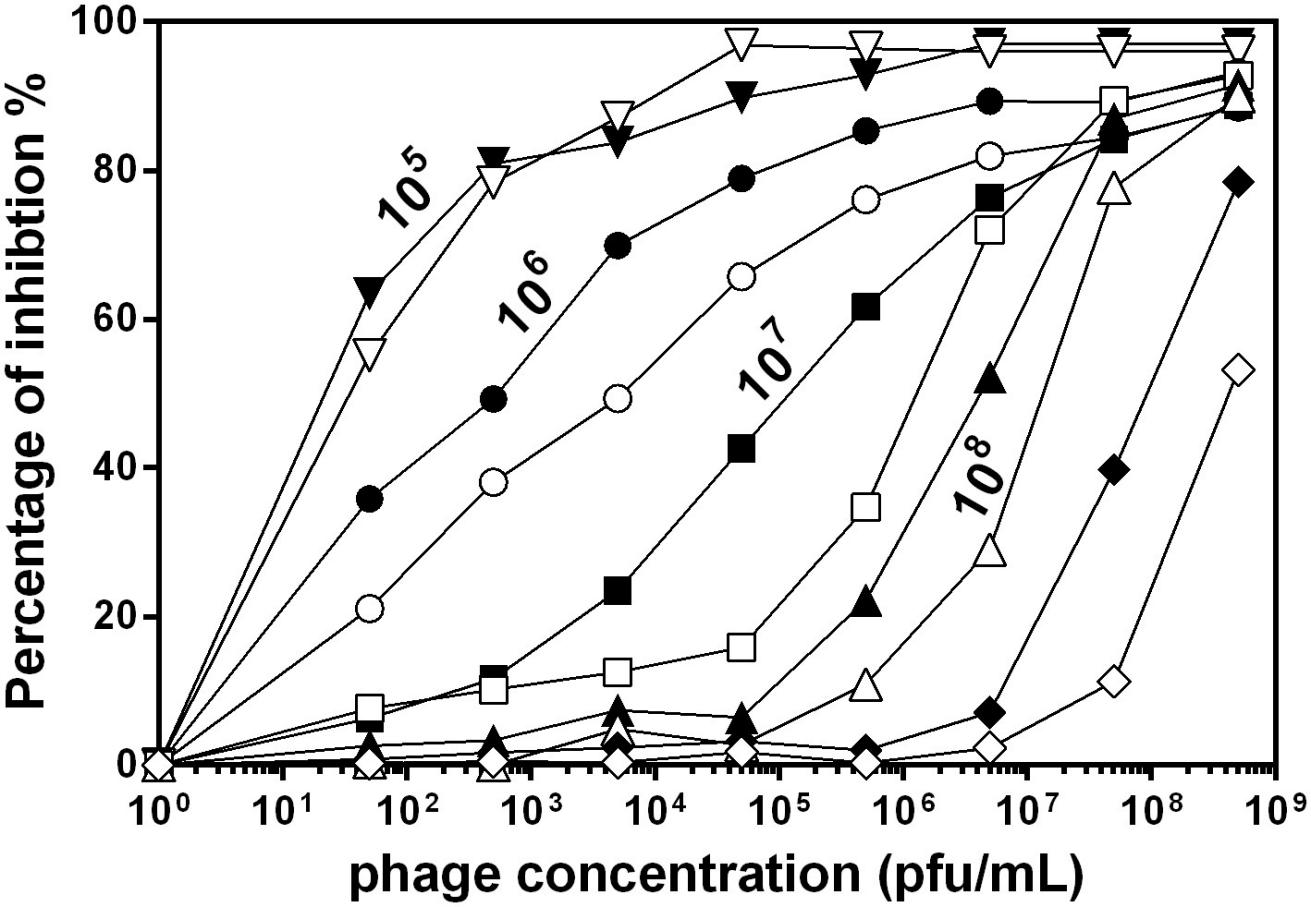
Percentage of inhibition as a function of phage concentration for different values of cell concentration. 5x108 (◇), 2.5x10^8^ (◆), 10^8^ (△), 5x10^7^ (▲), 2.5x10^7^ (□), 10^7^ (■), 5x10^6^ (○), 10^6^ (●), 5x10^5^ (▽), 10^5^ cfu/ml (▼). Percentages of inhibition were calculated as described in Materials and Methods.

In an attempt to make the assay quantitative, we used the procedure described in Materials and Methods to calculated the % inhibition (PI) caused by the presence of phages in each sample. The results, corresponding to each bacterial concentration, are presented in Fig 5 as a function of phage concentration. Fig 5 can be read as a set of calibration curves, each carried out at a different concentration of bacteria. In general, high bacterial concentrations are only sensitive to very high phage concentrations. At the same time, detection of low phage concentrations requires the use of low bacterial concentrations. To exemplify this, the calibration obtained with 10^8^ cfu/ mL provides a 3 log dynamic range between 5x10^5^ and 5x10^8^ pfu/mL. In the case of the curve obtained using 10^7^ cfu/mL, the dynamic range stretches 4 log between 5x10^2^ and 5x10^6^ pfu/mL. At 10^6^ cfu/mL, the sensitivity range narrows again to 3 log but allows detection of much lower phage concentration, between 5x10^1^ and 5x10^4^ pfu/mL.

Each of the points in the graph has been calculated from data of experiment carried out in triplicate. For each set of replicates, variability was always low with relative standard errors averaging 3.5% of the means. In order to see whether this experiments could be consistently reproduced the measurements corresponding to 10^7^ cells/mL were repeated three times at different dates using different inocula and different batches of culture medium and reagents. The results of these experiments allowed the estimation of an independent standard error for the measurements which has been included as a set of error bars for the 10^7^ cells/mL curve. In most cases standard errors are between 1 and 3% of the mean and, therefore, error bars are smaller than the symbols used in the graph. In two cases standard errors reach 4% of the mean and can be actually be seen as error bars expanding beyond the symbol. Overall, our conclusion is that the results are highly consistent and can be accurately reproduced in experiments carried out independently.

On the other hand, the detection limit of the method is inherently tied to the small volumes at which the assay is carried out. In a typical microplate assay, a working concentration of 50 pfu/mL (10 phages per microwell) in the microplate well requires taking 20 μL of a 500 pfu/mL sample in a total volume of 200 μL of phage + bacterial culture. The probability under these conditions of having a 20 μL sample containing zero phages, calculated using the probability mass function of the Poisson probability distribution (Material and methods, Eq 4), is 4.54x10^-5^ which means that only one out of approximately 22.000 samples will not contain phages. Lowering down the concentration to 5 phages per mL in the microplate well would require taking 20 μL of a 50 phage/μL sample in a total assay volume of 200 μL. Under these conditions the probability of having samples with no phages increases considerably, up to 0.368. At this probability practically 1 of every 3 samples would come void decreasing considerably the reliability of the assay.

Therefore, based on the design of the microplate assay and the volumes of sample involved, this method is able to detect 50 phages corresponding to an actual concentration of 500 phages/mL in the original sample. The time required for the assay under this conditions is 3.5 hours at the most, but this time can be reduced considerably when attempting to detect higher phage concentrations. Thus, detection of 10^8^ phages/mL can carried out in only 45 minutes.

In order to compare the method described in this paper with methods previously described in the literature, the performance of currently available methods, using nucleic acid detection, immunoassay, electron microscopy, impedance, SPR or release of intracellular components, has been summarized in Table 1. The sensitivity of these methods ranges across several orders of magnitude. At the low sensitivity end of the spectrum, electron microscopy provides precise quantification in a short time, but it requires high phage titers (≥ 10^7^ phages/mL) to provide reliable results. In addition, electron microscopy requires expensive equipment and highly skilled personnel, while providing a very low analytical throughput. At the other end, the highest sensitivity is found in methods that measure the release of intracellular components (ATP, ß-galactosidase, ß-glucuronidase) which allow the detection of 10^1^ phages/mL with short protocols requiring 2–3 hours of assay. The remaining methods have limits of detection in the 10^2^−10^3^ phages/mL range with time-to-result between 1 and 6 hours. The assay proposed in this paper fits in this middle segment. Using relatively simple equipment it is possible to detect 10^2^ phages/mL in 3.5 hours, a time that can be shortened considerably at the expense of increasing the limit of detection.

**Table 1:** Sensitivity, expressed as the limit of detection, and time required for detection, in different methods currently available for phage detection.

Method	Limit of detection (pfu/mL)	Time to detection (h)	Reference
OD kinetics	10^2^	0.75–3.5	this work
Surface Plasmon Resonance (SPR)	10^2^	3	[33]
Impendance measurements	10^2^	6	[35]
ß-glucuronidase release	10^1^	2.5	[13]
ß-galactosidase release	10^1^	2.5	[31]
ATP release	10^1^	3	[32]
DNA—qPCR	10^2^	2	[22]
DNA—qLAMP	10^3^	1	[40]
DNA—PCR	10^3^	4	[21]
Antibodies—Paper based ELISA	10^3^	2	[25]
Antibodies—Carbon nanotubes	10^3^	1	[41]
Fluorescence microscopy	10^2^	1	[30]
Transmission electron microscopy	10^7^	1	[42]

In this paper we do not describe a fully applicable method. The results obtained with our *E*. *coli*/T4 model system cannot be directly extrapolated to other bacteria/phage systems. But we establish a proof of concept that shows that kinetic-based methods can provide reliable phage detection and quantification in a reasonably short period of time. The paper also describes a methodology backed up by a very extensive data set, that can be used as a solid framework for the development of solutions to specific problems. Development of methods for the detection of phage levels in food preservation applications or in phage therapy, or detection of phages in industrial or environmental applications would demand an extensive full-fledged study requiring careful standardization, a characterization of the effect of the analytical matrix and taking into account the kinetics of the particular phage/host system that was beyond the scope of our work. The approach we propose is not devoid of problems. Samples containing toxic compounds might show inhibition in the absence of phages thus leading to false positive readings. Also, the samples could contain heterogeneous phage populations with very different infection kinetics, thus precluding accurate calibration and quantitative use, relegating the assay to a qualitative detection method. The effect of toxicity can be addressed, if required, by separately assessing toxicity or including phage-resistant organisms as controls. All of this elements, as mentioned above, are part of a specific method development and should be taken into account for each specific application.

In summary, this study presents a model based on the measurement of OD kinetics for phage enumeration and detection, using simple and inexpensive equipment. Although it uses non-sophisticated technology it has shown sensitivity and response time comparable to other high-end methods. Due to the simplicity of the technology and of the analytical steps involved, we anticipate that the system is susceptible of miniaturization and automation for high-throughput applications.

## Supporting information

S1 Fig Evolution of optical density during time in cultures of *E*.*coli* exposed to different T4 phage concentrations. Several bacteria concentrations: **A**) 5x10^8^, **B**) 2.5,10^8^, **C**) 10^8^, **D**) 5x10^7^, **E**) 2.5x10^7^ **F**) 10^7^ **G**) 5x10^6^, **H**) 10^6^, **I**) 5x10^5^ and **J**) 10^5^ were tested against different phage concentrations: 5x10^8^ (◇), 5x10^7^ (◆), 5x10^6^ (△), 5x10^5^ (▲), 5x10^4^ (□), 5x10^3^ (■), 5x10^2^ (○), 5x10^1^ pfu/ml (●) and without phages (×). Error bars represent the standard deviation (n = 3).
(TIF)

S1 Dataset Contains the optical density (OD) vs time data corresponding to 90 different phage/bacteria combinations. Data have been used to build the graphs in Supplemental S1 Fig. A subset of the data has been used for Fig 2 of the paper.
(XLSX)
