# Supplementary figures and images for: Fast phage detection and quantification: An optical density-based approach
Source: PLoS One. 2019 May 9;14(5):e0216292. doi: 10.1371/journal.pone.0216292 (PMC6508699; doi:10.1371/journal.pone.0216292)

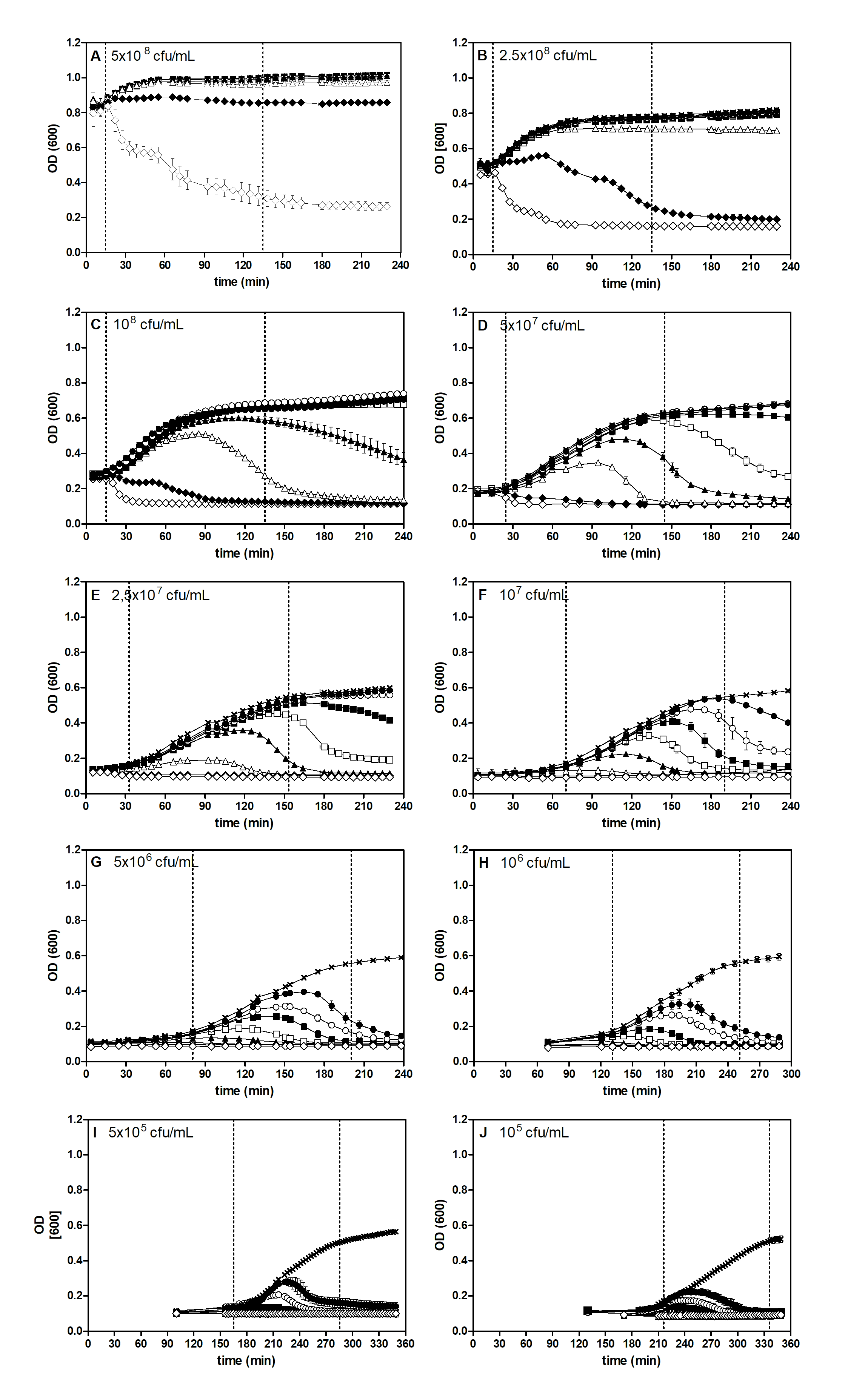

Supplement: S1 Fig — Several bacteria concentrations: A) 5x10^8^, B) 2.5,108, C) 108, D) 5x10^7^, E) 2.5x10^7^ F) 107 G) 5x10^6^, H) 106, I) 5x10^5^ and J) 105 were tested against different phage concentrations: 5x10^8^ (◇), 5x10^7^ (◆), 5x10^6^ (△), 5x10^5^ (▲), 5x10^4^ (□), 5x10^3^ (■), 5x10^2^ (○), 5x101 pfu/ml (●) and without phages (×). Error bars represent the standard deviation (n = 3). (TIF) [file pone.0216292.s001.tif]
